# Prevalence of *Angiostrongylus vasorum* in southern Belgium, a coprological and serological survey

**DOI:** 10.1186/s13071-016-1820-y

**Published:** 2016-10-06

**Authors:** Laetitia Lempereur, Ludovic Martinelle, Françoise Marechal, Calixte Bayrou, Anne Catherine Dalemans, Manuela Schnyder, Bertrand Losson

**Affiliations:** 1 Faculty of Veterinary Medicine, Center for Fundamental and Applied Research for Animal and Health (FARAH), Laboratory of Parasitology and Parasitic Diseases, University of Liège, Liège, Belgium; 2 Faculty of Veterinary Medicine, Experimental Station CARE – FePex, Center for Fundamental and Applied Research for Animal and Health (FARAH), University of Liège, Liege, Belgium; 3 Faculty of Veterinary Medicine, Center for Fundamental and Applied Research for Animal and Health (FARAH) Pathology Unit, University of Liège, Liège, Belgium; 4 Bayer Animal Health Benelux, Diegem, Belgium; 5 Institute of Parasitology, Vetsuisse Faculty, University of Zurich, Zurich, Switzerland

**Keywords:** *Angiostrongylus vasorum*, *Crenosoma vulpis*, Serology, Coprology, Dog, Belgium

## Abstract

**Background:**

Canine angiostrongylosis, a gastropod-borne helminthic infection, is increasingly being described in North America and is now reported in many European countries. In dogs*, Angiostrongylus vasorum* may cause a wide spectrum of clinical signs. Respiratory distress such as coughing and dyspnoea are the most frequently described manifestations. The aim of the present study was to gain additional information on the distribution, prevalence and risk factors associated with *A. vasorum* infection in dog from southern Belgium through the combined used of a commercially available in-clinic assay for detection of circulating antigen (Angio Detect™, IDEXX, Westbrook, USA) and coprology in two different canine populations: dogs with clinical signs compatible with angiostrongylosis and asymptomatic dogs or dogs presented for unrelated conditions (control).

**Results:**

A total of 979 dogs were enrolled in the study from November 2014 until February 2016. Seven hundred fifty-seven dogs were included in the control group, whereas 222 dogs had clinical signs compatible with angiostrongylosis. Forty-six dogs out of 979 (4.7 %) had *A. vasorum* circulating antigen. There was a highly significant difference between the two populations (3.6 % (27/747) and 8.6 % (19/222) in control and symptomatic dogs, respectively) (*P* = 0.00379). First stage larvae (L1) of *A. vasorum* were found in seven out of 24 serologically positive control dogs and in six out of 17 serologically positive symptomatic dogs. Interestingly, L1 of *Crenosoma vulpis* were detected by Baermann technique in one control and nine symptomatic dogs, respectively. Out of 17 Angio Detect™ (IDEXX, Westbrook, USA) positive dogs with negative (14) or not performed Baermann test (three), one dog was positive in both in-house ELISAs (Ag and Ab) and one dog was positive for Ag. Statistical analysis was unable to detect any risk factors associated with the direct and/or indirect detection of *A. vasorum*.

**Conclusions:**

This seroepidemiological study demonstrated for the first time a high seroprevalence in Southern Belgium for *A. vasorum*. The Angio Detect™ was found to be suitable in this context as the collection, preservation and examination of stools were difficult. Nevertheless, discrepancies were observed between the different available tests. Additional research is clearly needed. Also, coproscopy remains a very useful tool in dogs infected for less than nine weeks and for the identification of other canine lung nematodes such as *C. vulpis*. This study also demonstrates that asymptomatic dogs may shed *A. vasorum* L1 in their faeces and therefore contribute to the maintenance of *A. vasorum* life-cycle.

## Background

Canine angiostrongylosis, a gastropod-borne helminthic infection caused by the so-called “French heartworm”, was first reported in France in the 1800s [1]. The disease is increasingly being described in North America [2] and is reported now in many European countries [3]. The red fox (*Vulpes vulpes*) is considered as the main reservoir of this nematode [4] and many species of slugs and snails may act as intermediate hosts [5].

In dogs, *Angiostrongylus vasorum* may cause a wide spectrum of clinical signs from mild to severe, which can lead to death. Respiratory distress such as coughing and dyspnoea are the most frequently described manifestations. The diagnosis of the disease is sometimes very challenging as infected animals may exhibit clinical signs overlapping those of other conditions [6], be atypical such as bleeding and neurological disorders or be absent [7–9].

Despite the fact that epidemiological models indicate that Belgium has a highly favourable climate for the completion of *A. vasorum* life-cycle [10], the parasite was not recorded in this country until 2013 when a fatal autochthonous case was described in a four and a half month puppy [11]. Very recently, nine additional cases were diagnosed through Baermann faecal examination and quantitative PCR on bronchoalveolar lavage fluid [12, 13], which suggests that the parasite could be well established in Belgium at least in the study area (Wallonia, southern Belgium). However, it is well known that *A. vasorum* has a patchy distribution [14, 15] and this might be a challenge for the collection of reliable epidemiological data.

A confirmatory diagnosis can be obtained using the Baermann method, based on the isolation and microscopic identification of *A. vasorum* first stage larvae (L1) from faeces, showing the characteristic notch feature on the tail [16]. Nevertheless, the availability of newly developed commercial tests targeting circulating *A. vasorum* antigens has made possible the in-clinic diagnosis of this infection [17], while the ELISAs used for mass-screening in different canine populations allowed the implementation of region- or even nation-wide surveys [18–20]. The aim of the present study was to gain additional information on the distribution, prevalence and risk factors associated with *A. vasorum* infection in the dog through the combined used of in-clinic detection of circulating antigen and of coprology in two different canine populations: dogs with clinical signs compatible with angiostrongylosis and asymptomatic dogs or dogs presented for unrelated conditions.

## Methods

### Collection of samples and data

**Fig. 1:**
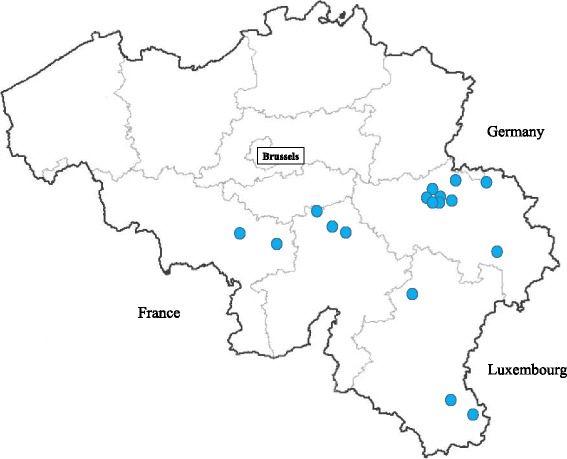
Selected veterinary clinics and their locations across southern Belgium

The survey was conducted from November 2014 until February 2016. Seventeen small animal practices were selected on voluntary basis across Southern Belgium (Wallonia) (Fig. 1). In each practice samples were collected from dogs belonging to two populations: a first random dog population (called “control” thereafter) presented for unrelated conditions (such as vaccination, spaying, traumas, skin conditions and others) whereas the second population included dogs showing clinical signs compatible with angiostrongylosis such as coughing, exercise intolerance, dyspnoea, bleeding or neurological disorders. Dogs from any breeds, age and sex were included in this survey. These two populations were selected based on the exclusion criteria of absence of travel history outside Belgium during the three previous months.

Simultaneously, a questionnaire was filled in by the owner: sex, age, breed, locality, life style and recent anti-parasitic treatments, if any, were recorded.

### Sample analysis

Blood samples from these dogs were collected in dry or heparinised tubes and centrifuged in order to obtain serum or plasma. An in-clinic serological test detecting *A. vasorum* circulating Ag (Angio Detect™, IDEXX, Westbrook, USA) was used for initial screening of all dogs (979) following manufacturer’s instructions, with a sensitivity of 84.6 % and a specificity of 100 %, as previously described [17]; reading of the test was performed strictly at 15 min. Intensity of positive results was assessed as +, ++, +++ [17]. All practices have been trained and monitored by the first author in order to obtain comparable results.

Recently passed stools from dogs with a positive serological screening were collected on 3 consecutive days. In some cases, this was not possible, or stools were obtained only once or twice. Additionally, some serologically negative dogs with a high suspicion of lungworm infection (showing dyspnoea or coughing or living with another seropositive dog) were also screened through coproscopy by isolation of L1 through Baermann larval migration-technique. This examination was done at the Laboratory of Parasitology of the Faculty of Veterinary Medicine, University of Liège. Differentiation between *A. vasorum* and *C. vulpis* L1 was performed based on morphological criteria [21].

Sera presenting conflicting results between serology and coprology were sent to the Institute of Parasitology, Vetsuisse Faculty, University of Zurich, Switzerland, and were further analysed for the presence of circulating *A. vasorum* antigens using monoclonal and polyclonal antibodies in a sandwich-ELISA, with a sensitivity of 95.7 % and a specificity of 94.0 %, as previously described [22]. Additionally, a sandwich-ELISA (sensitivity 81.0 %, specificity 98.8 %) using *A. vasorum* adult somatic antigen purified by monoclonal antibodies (mAb Av 5/5) was used for specific antibody detection [23]. All test runs included a background control, a conjugate control, three positive control sera from three experimentally infected dogs and two negative control sera from uninfected dogs.

### Statistical analysis

To identify possible risk factors associated with the seropositivity to *A. vasorum*, answers to the questionnaire were encoded and merged with the serological results (positive, negative) of each dog. The host variables were made available to the model, with sex as a binary variable (male or female), access to garden, terrace, urban park, forest, street and vegetable garden as binary variable (yes/no), housing as a binary variable (inside or access to the outside), clinical signs compatible with angiostrongylosis as a binary variable (yes/no), age as a categorical variable (< 1 year, between 1 and 3 years, between 3 and 8 years and over 8 years), post code of the owner’s domicile as a categorical variable and locality as a categorical variable (urban, rural or periurban).

An univariate analysis was conducted and odds ratio's (OR) with 95 % confidence intervals (CIs 95 %) were attributed to each variable. All variables with *P* < 0.20 in univariate analysis were then included in subsequent multivariate logistic regression analyses. A *P* value < 0.05 was used to define statistical significance.

Statistical analyses were performed using the R software/environment (R-3.1.2, R Foundation for Statistical Computing, http://www.r-project.org/) and SAS software, Version 9.3 TS level 1 M2 of the SAS System for Unix and SAS University Edition (SAS Institute, Cary, NC, USA).

## Results

A total of 979 dogs were enrolled in the study. Seven hundred fifty-seven were included in the control group, whereas 222 dogs had clinical signs compatible with angiostrongylosis. The distribution of samples according to the different tests (serology versus coprology) is given in Table 1. Forty-six dogs out of 979 (4.7 %) had *A. vasorum* circulating antigen detected with the rapid in-clinic assay. There was a highly significant difference between the two populations (3.6 % (27/757) and 8.6 % (19/222) in control and symptomatic dogs respectively) (*χ* ^*2*^ = 8.4702, *df* = 1, *P* = 0.00361).

**Table 1:** Results of Angio Detect™ (IDEXX, Westbrook, USA) rapid assay test performed with sera of symptomatic and control dogs. Baermann coprological analysis was performed on dogs positive for Angio Detect™ test or suspected of angiostrongylosis

	Parasite	Control dogs *n* = 757 (%)	Symptomatic dogs *n* = 222 (%)	Total *n* = 979 (%)
Total Angio Detect™-positive		27/757 (3.6)	19/222 (8.6)	46/979 (4.7)
Angio Detect™-positive and Baermann not performed		3/27	2/19	5/46
Angio Detect™-positive and Baermann-positive	L1 *A. vasorum*	7/24 (29)	6/17 (35)	13/41 (32)
	L1 *C. vulpis*	0/24	1/17	1/41
	L1 *A. vasorum* and *C. vulpis*	0/24	2/17	2/41
Angio Detect™-positive and Baermann-negative		17/24	8/17	25/41
Angio Detect™-negative and Baermann-positive	L1 *A. vasorum*	0/1	0/5	0/6
	L1 *C. vulpis*	1/1	4/5	5/6
	L1 *A. vasorum* and *C. vulpis*	0/1	1/5	1/6

**Fig. 2:**
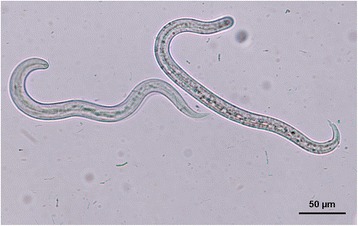
First stage larvae of *Crenosoma vulpis* (left) and *Angiostrongylus vasorum* (right) found in faeces of a dog isolated by the Baermann method

Stools were obtained from 41 seropositive dogs (24 and 17 in control and symptomatic dogs respectively) and 6 seronegative dogs with strong suspicion of angiostrongylosis (1 and 5 in control and symptomatic dogs respectively). Stools from 5 seropositive dogs were not available for testing. L1 of *A. vasorum* were found in 7 out of 24 (29 %) serologically positive (Angio Detect™) control dogs and in 6 out of 17 (35 %) serologically positive symptomatic dogs. Interestingly L1 of *C. vulpis* were detected by the larval migration Baermann technique in 1 control and 9 symptomatic dogs, respectively. In the latter group 1 dog was found seropositive for *A. vasorum* but only *C. vulpis* L1 were found, and 1 dog was found seronegative for *A. vasorum* but L1 of *A. vasorum* and *C. vulpis* were found *via* the Baermann technique (Fig. 2). A majority of seropositive (Angio Detect™) and symptomatic dogs exhibited cardio-pulmonary symptoms (cough, dyspnoea and exercise intolerance). Neurological signs and bleeding were recorded in one symptomatic dog (Table 2).

**Table 2:** Number of dogs showing different clinical signs recorded by the veterinarians in the questionnaire

Clinical signs	Dogs	Angio Detect™-positive
Coughing	86	6
Dyspnoea	19	3
Exercise intolerance	39	3
Bleeding	11	1
Neurological disorders	13	1
Coughing + exercise intolerance	44	5
Coughing + bleeding	6	0
Bleeding + neurological disorders	1	0
No clear statement	3	0
Total	222	19

Eighteen sera of dogs with conflicting results between serology and coproscopy (or coproscopy not performed) were analysed by in-house ELISAs at the Institute of Parasitology of the University of Zurich, Switzerland. Out of 17 seropositive dogs with Angio Detect™ and with negative or not performed Baermann test, one dog was positive for both ELISAs (Ag and Ab) and one dog was positive for Ag ELISA only. The dog found seronegative for *A. vasorum* with Angio Detect™ but positive for L1 of *A. vasorum* and *C. vulpis* was found positive by Ab ELISA (Table 3).

**Table 3:** Results of in-house ELISAs (Ag and Ab) tested on 18 dogs with conflicting results between serology and coprology (negative) or with coprology not performed

	Angio Detect™-positive				Angio Detect™-negative			
	ELISA Ag		ELISA Ab		ELISA Ag		ELISA Ab	
	No. positive/ No. tested)	No. negative/ No. tested	No. positive/No. tested	No. negative/ No. tested	No. positive/ No. tested	No. negative/ No. tested	No. positive/ No. tested	No. negative/ No. tested
Symptomatic	1/4	3/4	1/4	3/4	0/1	1/1	1/1	0/1
Control	1/13	12/13	0/13	13/13	–	–	–	–
Total	2/17	15/17	1/17	16/17	0/1	1/1	1/1	0/1

**Fig. 3:**
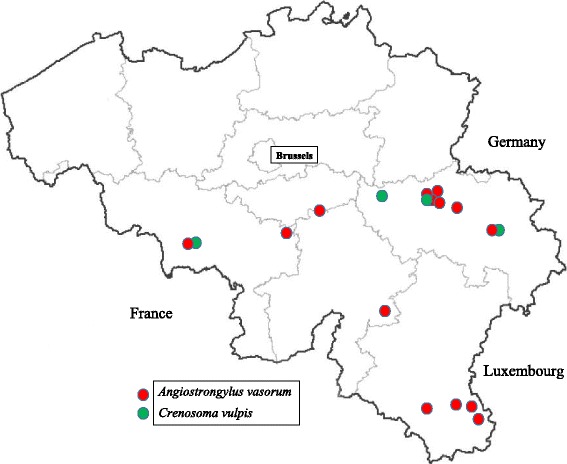
Locations (based on postal codes) of seropositive dogs for *A. vasorum* according to Angio Detect™ (IDEXX, Westbrook, USA) and *C. vulpis* according to Baermann examination in southern Belgium

Data obtained from the questionnaire provided the different localities where dogs were living (Fig. 3). In the univariate analysis access to the forest (*Z* = -1.673, *P* = 0.094) and clinical signs (*Z* = 2.895, *P* = 0.00379) were associated with seropositivity to *A. vasorum.* These two explanatory variables were included in the multivariate model. However, only the presence of clinical signs compatible with angiostrongylosis resulted as a risk factor for *A. vasorum* seropositivity (*P* = 0.0079; OR = 3.1; 95 % CI: 1.33–7.22).

## Discussion

Originally, three practices were chosen for a pilot study because of previously diagnosed canine angiostrongylosis in the region. Additionally, 14 other practices in southern Belgian were enrolled in the study based on the interest they showed to test and deliver samples in order to explore whether more cases could be diagnosed. The implementation of this type of survey relies heavily on the willingness and rigour of the different participants, namely the veterinary practices. This may be considered as a potential bias. However, canine practices were selected in the five different provinces of southern Belgium (Wallonia) where a large number of both symptomatic and control dogs were sampled. Therefore, the present survey, the first large scale epidemiological study conducted in Belgium, should be considered as a pilot study aiming to give a fairly good estimation of the impact of angiostrongylosis in the canine population of southern Belgium.

For practical reasons (ease of use and rapidity) the 979 canine sera were screened using an in-clinic test (Angio Detect™) which is able to detect specific *A. vasorum* circulating antigen as early as nine weeks post-experimental inoculation. This test has a good sensitivity (84.6 %) for clinically affected dogs, and a very high specificity (100 %) regarding different lungworms [17]. In total 46 dogs (4.7 %) had specific circulating antigen which indicates that *A. vasorum* infections is well established in the canine southern Belgian population. This is in agreement with recently published data dealing with single case reports [11, 13] or small clinical series [12]. Large-scale seroepidemiological surveys were previously performed in Europe using ELISAs, showing antigen detection in 0.5 to 2.17 % of the study populations [18–20, 24–26]. Among Belgium’s neighbouring countries, in Germany, 4003 randomly selected canine sera were collected from western federal states and tested by Schnyder et al. [18] using Ab and Ag ELISAs, the latter test being able to detect *A. vasorum* specific antigen as early as seven weeks post-infection [27]. A total of 20 sera (0.5 %) were antigen positive in that study. The comparison of this seroprevalence with the results of the control group presented in our study (3.6 %) confirms that the investigated area, which has a border with North Rhine Westphal and Rhineland-Palatinate must be very suitable for the completion of *A. vasorum* life-cycle [10], particularly considering that the rapid in-clinic assay is recommended for testing clinically affected dogs and less sensitive than the ELISAs that have been used in the mentioned study from Germany. In symptomatic dogs the proportion of antigen positive dogs was even higher (8.6 %). The majority of these dogs had cardio-pulmonary symptoms (cough, dyspnoea, exercise intolerance) which are by far the most commonly observed clinical signs in dogs with canine angiostrongylosis [9]. Coagulopathies and neurological disorders, which were only reported in one seropositive dog during the present study, are reported in canine angiostrongylosis but represent a small proportion of the patients [7].

Other epidemiological studies based on coproscopy confirmed that asymptomatic dogs can excrete L1 and thus contribute to the dissemination of the parasite in the environment. For example, in UK, Morgan et al. [28] found *A. vasorum* L1 in the faeces of 15 % and 2 % of symptomatic and asymptomatic dogs, respectively. Barutzki & Schaper [29] in Germany isolated L1 in 6 % of symptomatic dogs (*n* = 810) versus 0.1 % in a survey conducted earlier by the same authors in asymptomatic individuals [30]. It is not known whether these asymptomatic carriers may or may not develop clinical signs later on, as the majority of these dogs may have been treated with an anthelmintic drug.

The larval migration-technique was difficult to implement during this study: a total of 47 stool samples were obtained and in some cases, samples from only one day of collection were provided by the owners, or the stools had dried up. This might explain at least partly the high level of discrepancy observed between the serological assay (positivity) and the Baermann technique (negativity). Additional explanations could be linked to the technical characteristics of the in-clinic test which requires reading after 15 min sharp (and resulting in false positive results if reading is performed later), and the potential of non-reported anti-parasitic treatment by the owner (resulting in negative coproscopy while serology may still be positive) [27]. Indeed, in experimentally infected dogs, patency started between 7–8 weeks after infection, approximately at the same time of antigen detection (while antibody detection may start as soon as three weeks after infection), with the difference that dogs, once diagnosed seropositive, remain positive unless treated [27], in opposition to Baermann examination, where intermittent larval shedding can be observed [31]. The potential role of the immune response that inhibits the production of L1 as described in canine dirofilariosis [32] could also be hypothesised. Nevertheless, some discrepancies were not explained by the above hypotheses and some dogs positive with Angio Detect™ were not confirmed by in house ELISAs (Ag and/or Ab).

Interestingly, L1 of *C. vulpis* were observed in nine and one symptomatic and control dogs, respectively confirming the presence of this nematode in Belgium, as recently reported [33]. Consequently, this parasitic infection must be included in the differential diagnosis of pulmonary conditions. It is noteworthy that in five of these *C. vulpis* infected dogs the Angio Detect™ assay resulted in a negative result, while a mixed infection in these dogs may have been missed. This confirmed the high specificity of the test as previously demonstrated by Schnyder at al. [20] towards *C. vulpis* and other canine nematodes. In one symptomatic Angio Detect™ negative dog, L1 of both *A. vasorum* and *C. vulpis* were observed, whereas the sample was found to be positive with Ab ELISA. A possible explanation could be the production of immune complexes, as shown for *Dirofilaria immitis,* which could block Ag detection on commercially available tests [34, 35].

## Conclusions

In conclusion, this seroepidemiological study demonstrated a high seroprevalence in southern Belgium for *A. vasorum* and further investigation is necessary to check if this patchy distribution in the investigated area is also present in the northern part of Belgium. The Angio Detect™ was found to be suitable in this context as the collection, preservation and examination of stools were challenging. More research is needed in order to understand variability in the results obtained from different diagnostic tools. However, coproscopy remains a very useful tool in patent dogs infected for less than nine weeks and for the identification of other canine lung nematodes such as *C. vulpis* as mixed infections with different lungworm species may occur. This study also demonstrates that asymptomatic dogs or dogs presented for unrelated conditions may shed *A. vasorum* L1 in their faeces and therefore contribute to the maintenance of *A. vasorum* life-cycle. Consequently, practitioners in endemic areas should be sensitized and may suggest to screen for *A. vasorum* infection even in asymptomatic dogs. For dogs with eating behaviour at high-risk and potential ingestion of snails, regular testing or preventative monthly treatments are recommended [36].

## Abbreviations

Ab: Antibody
Ag: Antigen
ELISA: Enzyme-linked immunosorbent assay
L1: First-stage larvae

## References

[1] Serres E (1854). Entozoaires trouvés dans l’oreille droite, le ventricule correspondant et l’artère pulmonaire d’un chien. J Vétérinaires du Midi..

[2] Conboy GA (2011). Canine angiostrongylosis: the French heartworm: an emerging threat in North America. Vet Parasitol..

[3] Helm JR, Morgan ER, Jackson MW, Wotton P, Bell R (2010). Canine angiostrongylosis: an emerging disease in Europe. J Vet Emerg Crit Care..

[4] Tolnai Z, Szell Z, Sreter T (2015). Environmental determinants of the spatial distribution of *Angiostrongylus vasorum*, *Crenosoma vulpis* and *Eucoleus aerophilus* in Hungary. Vet Parasitol..

[5] Ferdushy T, Kapel CM, Webster P, Al-Sabi MN, Gronvold J (2009). The occurrence of *Angiostrongylus vasorum* in terrestrial slugs from forests and parks in the Copenhagen area, Denmark. J Helminthol.

[6] Di Cesare A, Traversa D, Manzocchi S, Meloni S, Grillotti E, Auriemma E (2015). Elusive *Angiostrongylus vasorum* infections. Parasit Vectors..

[7] Chapman PS, Boag AK, Guitian J, Boswood A (2004). *Angiostrongylus vasorum* infection in 23 dogs (1999–2002). J Small Anim Pract..

[8] Colella V, Lia RP, Premont J, Gilmore P, Cervone M, Latrofa MS (2016). *Angiostrongylus vasorum* in the eye: new case reports and a review of the literature. Parasit Vectors..

[9] Koch J, Willesen JL (2009). Canine pulmonary angiostrongylosis: an update. Vet J.

[10] Morgan ER, Jefferies R, Krajewski M, Ward P, Shaw SE (2009). Canine pulmonary angiostronylosis: the influence of climate on parasite distribution. Parasitol Int..

[11] Jolly S, Poncelet L, Lempereur L, Caron Y, Bayrou C, Cassart D (2014). First report of a fatal autochthonous canine *Angiostrongylus vasorum* infection in Belgium. Parasitol Int..

[12] Canonne A-M, Roels E, Caron Y, Losson B, Bolen G, Peeters D (2015). Detection of *Angiostrongylus vasorum* by quantitative PCR in bronchoalveolar fluid in Belgian dogs. J Small Animal Practice..

[13] Sarre C, Willems A, Daminet S, Claerebout E (2015). Autochthonous *Angiostrongylus vasorum* infection in a Border collie in Belgium. Vlaams Diergeneeskundig Tijdschrift..

[14] Bolt G, Monrad J, Koch J, Jensen A (1994). Canine angiostrongylosis: a review. Vet Rec..

[15] Blehaut TR, Hardstaff JL, Chapman PS, Pfeiffer DU, Boag AK, Guitian FJ (2014). Spatial, demographic and clinical patterns of *Angiostrongylosis vasorum* in the dog population of Southern England. Vet Rec..

[16] Guilhon J, Cens B (1973). *Angiostrongylus vasorum* (Baillet, 1866): Étude biologique et morphologique. Ann Parasitol Hum Comp..

[17] Schnyder M, Stebler K, Naucke TJ, Lorentz S, Deplazes P (2014). Evaluation of a rapid device for serological in-clinic diagnosis of canine angiostrongylosis. Parasit Vectors..

[18] Schnyder M, Schaper R, Bilbrough G, Morgan ER, Deplazes P (2013). Serepidemiological survey for canine angiostrongylosis in dogs from Germany and the UK using combined detection of *Angiostrongylus vasorum* antigen and specific antibodies. Parasitology..

[19] Schnyder M, Schaper R, Pantchev N, Kowalska D, Szwedko A, Deplazes P (2013). Serological detection of circulating *Angiostrongylus vasorum* antigen and parasite–specific antibodies in dogs from Poland. Parasitol Res.

[20] Lurati L, Deplazes P, Hegglin D, Schnyder M (2015). Seroepidemiological survey and spatial analysis of the occurrence of *Angiostrongylus vasorum* in Swiss dogs in relation to biogeographic aspects. Vet Parasitol..

[21] McGarry JW, Morgan ER (2009). Identification of first-stage larvae of metastrongyles from dogs. Vet Rec..

[22] Schnyder M, Tanner I, Webster P, Barutzki D, Deplazes P (2011). An ELISA for sensitive and specific detection of circulating antigen of *Angiostrongylus vasorum* in serum samples of naturally and experimentally infected dogs. Vet Parasitol..

[23] Schucan A, Schnyder M, Tanner I, Barutzki D, Traversa D, Deplazes P (2012). Detection of specific antibodies in dogs infected with *Angiostrongylus vasorum*. Vet Parasitol..

[24] Guardone L, Schnyder M, Macchioni F, Deplazes P, Magi M (2013). Serological detection of circulating *Angiostrongylus vasorum* antigen and specific antibodies in dogs from central and northern Italy. Vet Parasitol.

[25] Schnyder M, Schaper R, Lukács Z, Hornok S, Farkas R (2015). Combined serological detection of circulating *Angiostrongylus vasorum* antigen and parasite-specific antibodies in dogs from Hungary. Parasitol Res.

[26] Alho AM, Schnyder M, Schaper R, Meireles J, Belo S, Deplazes P (2016). Seroprevalence of circulating *Angiostrongylus vasorum* antigen and parasite-specific antibodies in dogs from Portugal. Parasitol Res.

[27] Schnyder M, Jefferies R, Schucan A, Morgan ER, Deplazes P (2015). Comparison of coprological, immunological and molecular methods for the detection of dogs infected with *Angiostrongylus vasorum* before and after anthelmintic treatment. Parasitology..

[28] Morgan ER, Jefferies R, van Otterdijk L, McEnirya RB, Allena F, Bakewella M (2010). *Angiostrongylus vasorum* infection in dogs: presentation and risk factors. Vet Parasitol..

[29] Barutzki D, Schaper R (2009). Natural infection of *Angiostrongylus vasorum* and *Crenosoma vulpis* in dogs in Germany (2007–2009). Parasitol Res..

[30] Barutzki D, Schaper R (2003). Endoparasites in dogs and cats in Germany 1999–2002. Parasitol Res..

[31] Oliveira-Junior SD, Barcante JM, Barcante TA, Dias SR, Lima WS (2006). Larval output of infected and re-infected dogs with *Angiostrongylus vasorum* (Baillet, 1866) Kamensky, 1905. Vet Parasitol..

[32] Traversa D, Di Cesare A, Conboy G (2010). Canine and feline cardiopulmonary parasitic nematodes in Europe: emerging and underestimated. Parasit Vectors..

[33] Caron Y, Merveille A-C, Losson B, Billen F (2014). *Crenosoma vulpis* infection in two young dogs in Belgium. Vet Rec Case Reports..

[34] Drake J, Gruntmeir J, Merritt H, Allen L, Little S (2015). False negative antigen tests in dogs infected with heartworm and placed on macrocyclic lactone preventives. Parasit Vectors..

[35] Little S, Raymond M, Thomas J, Gruntmair J, Hostetler J, Meinkoth J (2015). Heat treatment prior to testing allows detection of antigen of *Dirofilaria immitis* in feline serum. Parasit Vectors..

[36] ESCCAP, worm control in dogs and cats. 2010; guidelines 01 second edition. Malvern: ESCCAP.

